# Predictive Value of Motor Evoked Potentials in the Resection of Intradural Extramedullary Spinal Tumors in Children

**DOI:** 10.3390/jcm12010041

**Published:** 2022-12-21

**Authors:** Lukasz Antkowiak, Monika Putz, Ryszard Sordyl, Szymon Pokora, Marek Mandera

**Affiliations:** Department of Pediatric Neurosurgery, Medical University of Silesia in Katowice, 40-752 Katowice, Poland

**Keywords:** intraoperative neurophysiological monitoring, motor evoked potentials, intradural extramedullary spinal tumor, pediatric, neurological deficits

## Abstract

This study aimed to evaluate the predictive value of motor evoked potentials (MEP) in the resection of pediatric intradural extramedullary (IDEM) tumors. Additionally, we aimed to assess the impact of MEP alerts on the extent of tumor resection. Medical records of pediatric patients who underwent resection of IDEM tumors with the assistance of MEP between March 2011 and October 2020 were reviewed. The occurrence of postoperative motor deficits was correlated with intraoperative MEP alerts. Sixteen patients were included. MEP alerts appeared in 2 patients (12.5%), being reflective of new postoperative motor deficits. Among the remaining 14 patients without any intraoperative MEP alerts, no motor decline was found. Accordingly, MEP significantly predicted postoperative motor deficits, reaching sensitivity, specificity, positive predictive value (PPV), and negative predictive value (NPV) of 100% (*p* < 0.001). In the absence of MEP alerts, 11 out of 14 patients (78.6%) underwent GTR, while no patient with intraoperative IONM alerts underwent GTR (*p* = 0.025). Although MEP alerts limit the extent of tumor resection, the high sensitivity and PPV of MEP underline its importance in avoiding iatrogenic motor deficits. Concurrently, high specificity and NPV ensure safer tumor excision. Therefore, MEP can reliably support surgical decisions in pediatric patients with IDEM tumors.

## 1. Introduction

Spinal canal tumors account for 5–10% of all central nervous system (CNS) tumors in children, with approximately 25% of them being classified as intradural extramedullary (IDEM) tumors [1,2]. The most common tumors in this location are metastases from primary brain tumors and nerve sheath tumors, including schwannomas and neurofibromas [3]. Surgical procedures should aim at gross total resection (GTR) in each case. While GTR is considered curative in the majority of patients, it may be achieved with an inadvertent sacrifice of spinal cord integrity, resulting in postoperative neurological decline [4]. Through the utilization of intraoperative neurophysiological monitoring (IONM), the integrity of both ascending and descending white matter tracts can be successfully evaluated during the entire surgical procedure. Among various IONM modalities, somatosensory evoked potentials (SSEP) serve as an indicator of sensory pathways (specifically dorsal column) integrity, while motor-evoked potentials (MEP) and D-waves are intended for motor pathways monitoring [5,6,7,8,9]. In case of significant IONM changes occurring during tumor removal, the surgeon is alerted of the occurrence of potential neurological decline when the tumor removal is continued at that stage. In that way, precautious measures can be undertaken to stop the neurological injury at the reversible state, thus preventing permanent postoperative neurological deficits. While the efficacy of IONM in spinal surgery and intramedullary spinal cord tumor (IMSCT) removal has already been established and considered the standard of care [10,11,12,13,14,15,16], its value in IDEM tumors remains still under debate. Although growing evidence progressively supports the utility of IONM to predict postoperative neurological deficits [4,17,18,19,20,21], no guidelines exist on its routine implementation in IDEM surgery. On account of that, the utilization of IONM in IDEM surgery depends on the institution’s experience and preferences rather than evidence-based recommendations.

Moreover, the literature addressing the usefulness of IONM solely in pediatric spinal tumors remains sparse [22,23,24]. In our previous study on pediatric IMSCTs, we successfully proved that D-wave and MEP significantly predicted postoperative motor deficits, while SSEP failed to achieve sufficient accuracy for the prediction of sensory deficits [22]. However, to date, no study reported the utility of IONM in pediatric IDEM tumors. Therefore, the purpose of our study was to evaluate the predictive value of MEP in the resection of pediatric IDEM tumors. Additionally, we aimed to assess the impact of IONM alerts on the extent of tumor resection.

## 2. Materials and Methods

We retrospectively reviewed the medical records of pediatric patients (≤18 years old at admission) who underwent resection of IDEM tumor with the assistance of MEP between March 2011 and October 2020 at the Department of Pediatric Neurosurgery, Medical University of Silesia in Katowice. Only patients with complete clinical and IONM data were included. The following data were extracted: patient demographics, preoperative and postoperative clinical status, tumor location in relation to the spinal cord, tumor span, the extent of resection (EOR), intraoperative MEP changes, and tumor histology. All patients underwent surgery under total intravenous anesthesia (TIVA), with the same conditions as described in our previous study [22].

### 2.1. Clinical Evaluation

The neurological examination was conducted routinely in all patients both at admission and at discharge from the hospital using the modified McCormick Scale. Patients whose preoperative McCormick grade decreased following surgery were considered as improved. When the patient’s postoperative McCormick grade was identical to the preoperative state, the patient was evaluated as unchanged. Finally, patients with increased postoperative McCormick grade, as compared to the preoperative McCormick grade, were considered as neurologically worsened. Additionally, patients who presented with postoperative worsening of their neurological status were followed-up in order to determine whether their deficits were transient or persistent.

### 2.2. Motor Evoked Potentials

ISIS Xpert Plus System (Inomed Polska, Rokitnica, Poland) was used for all MEP recordings. Subdermal corkscrew electrode placement was consistent with the international 10–20 system. C1/C2 electrodes were placed in order to evoke responses from the lower extremities, while C3/C4 electrodes were applied to the upper extremities. Routinely, biceps brachii, thenar muscle, quadriceps femoris, and triceps surae muscles were monitored. MEPs were elicited via a train of 4–6 electrical pulses (intensity between 90 and 140 mA, pulse duration 500 μs). The frequency of the MEP monitoring depended on the surgeon’s preferences and the stage of the surgery. A decrease in MEP amplitude of >50% was considered a warning criterion, which imposed a temporary stop of surgery. Firstly, the hypotension was corrected by the anesthesiologist. Then the surgical field was poured with warm saline. In case of persistent decrease > 50% of MEP amplitude, further surgical resection was abandoned.

### 2.3. Statistical Analysis

The sensitivity, specificity, positive predictive value (PPV), and negative predictive value (NPV) of MEP for postoperative motor deficits were calculated. Mann–Whitney test was applied for the comparison of continuous variables, while the chi-square test was used for discrete variables. The accuracy of MEP to predict postoperative motor deficits was calculated via area under the receiver operating characteristic (AUROC) curve. Statistica 13.3 software (StatSoft Polska, Krakow, Poland) was used for all statistical analyses. Values were considered statistically significant at *p* < 0.05. The study power in our calculations amounted to 0.81.

## 3. Results

A total of 16 consecutive pediatric patients were included, with an average age of 10.9 years (range 1–17.7 years). Most patients presented with motor deficits (*n* = 10, 62.5%), followed by sensory deficits (*n* = 6, 37.5%) and urinary incontinence (*n* = 2, 12.5%). Five patients were neurologically intact at admission (31.5%). Detailed patient characteristics are presented in Table 1.

Ependymoma constituted the most common histological tumor type, identified in 6 patients (37.5%), followed by meningioma in 3 individuals (18.8%), schwannoma (6.3%), neurenteric cyst (6.3%), dermoid cyst (6.3%), epidermal cyst (6.3%), and lipoma (6.3%), occurring in one patient each. Two patients presented with metastatic IDEM tumors, one being identified as medulloblastoma (6.3%), while the other one as a nongerminous germ-cell tumor (NGGCT) (6.3%).

GTR was performed in 11 patients (68.8%), while 5 remaining individuals underwent subtotal tumor resection (31.2%). At the discharge, 4 patients improved (25%) neurologically, 10 remained stable (62.5%), and 2 patients experienced a neurological decline (12.5%).

Intraoperative MEP alerts appeared in 2 patients (12.5%), which correlated with the occurrence of new postoperative motor deficits (PPV = 100%). Both patients died shortly after discharge due to the tumor progression. Therefore, their neurological deficits persisted. No false-positive MEP alerts were identified (NPV = 100%). Among the remaining 14 patients without any intraoperative MEP alerts, no motor function decline was found. With an overall 100% sensitivity and specificity, MEP significantly predicted the occurrence of new postoperative motor deficits (*p* < 0.001, AUROC = 1).

### The Impact of MEP on the Extent of Tumor Resection

**Figure 1:**
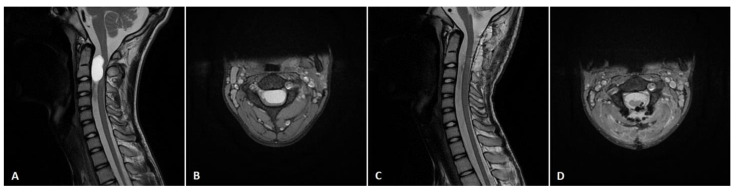
An exemplary case of a 14-year-old boy (patient no. 16), who presented with a left-sided motor deficit in the upper limb. Preoperative T2 (**A**) sagittal and (**B**) axial MRI scan showed C1-C2 intradural extramedullary tumor. The patient underwent gross-total tumor resection via the posterior approach with laminectomy. Postoperative T2 (**C**) sagittal and (**D**) axial MRI scan confirmed complete tumor removal. Postoperatively, patients’ motor function improved.

In the absence of MEP alerts, 11 out of 14 patients (78.6%) underwent GTR, while no patient with intraoperative IONM alerts underwent GTR. Consequently, we found that IONM alerts significantly affected the EOR (*p* = 0.025). An exemplary case of a patient who underwent GTR without MEP alerts is presented in Figure 1.

## 4. Discussion

While the role of IONM in adult IDEM tumor surgery has already been documented, to date, no study addressed the utility of IONM exclusively in the pediatric cohort. It seems crucial to specifically define the application of IONM in children due to the neurophysiological disparity between adults and children. Several factors are known to influence MEP reliability, including anesthetics, baseline neurological status, and corticospinal tract myelination [25,26]. While the immaturity of corticospinal tracts before 2 years of age makes MEPs difficult to obtain, it has already been revealed that MEPs can be acquired even in infants between 1 and 3 months of age [27]. However, younger patients still need a higher threshold voltage for MEP to be elicited [28]. Taking into consideration all the difficulties and disparities in the pediatric IONM compared to adults, in the present study, we aimed to evaluate the feasibility and reliability of MEP in managing pediatric IDEM tumors. It is noteworthy that our study’s cohort slightly differs regarding the histological distribution of tumors, compared to the typical pediatric IDEM tumor distribution. While generally, the majority of pediatric IDEM tumors constitute meningiomas, schwannomas, and neurofibromas [29], we observed the predominance of ependymomas (37.5%) followed by meningiomas (18.8%). Notably, although ependymoma is perceived as an intramedullary tumor, the ependymomas presented in our series were mainly (66.7%) myxopapillary ependymomas, being IDEM tumors with a tendency to occur in the lumbosacral area [30]. Additionally, six out of sixteen tumors in our cohort were located below the conus medullaris. It is apparent that the risk profile differs between the surgery of a tumor at the level of spinal cord and one located below the conus medullaris. Although the extent of iatrogenic deficits varies between those two locations, the interpretation of MEP alerts in our study remained similar. In case of significant MEP decline, the surgery was temporarily stopped in that area, mean arterial pressure was maintained above 90 mmHg and the surgical field was poured with warm saline. These preventive measures aimed to avoid injury to the motor pathway. If MEP values returned to normal range, the surgery was continued.

All patients included in our study had successfully elicited baseline MEPs, which were further monitored during the entire surgery, with the frequency depending on the surgeon’s preferences. Two patients showed intraoperative >50% MEP amplitude decrease, which did not improve following preventive measures, leading to the definitive interruption of surgery, thus resulting in subtotal tumor removal in those cases. In the postoperative period, both patients presented with significant worsening of their motor function. These patients did not undergo follow-up since the initial tumor aggressiveness resulted in patients’ death in a few months following surgery. Therefore, we could not evaluate the long-term persistence of their deficits. Among the remaining 14 individuals, neither significant IONM changes nor new postoperative motor deficits occurred. Accordingly, we found MEP of 100% sensitivity, specificity, PPV, and NPV, thus being significantly predictive of the presence of postoperative motor decline (*p* < 0.001). Despite being promising, our data are based on a limited cohort and a small number of IONM alerts which could have artificially increased the MEP accuracy values. However, the power of our study amounted to 0.81, thus supporting the validity of our data. Nevertheless, we presume that with a larger cohort and more IONM events, in particular, sensitivity and PPV would be expected to decrease as a result of the increase in positive IONM alerts, including false-positive ones.

Following the successful implementation of the IONM into the routine surgical procedures of IMSCTs removal, growing evidence increasingly supports its efficacy in IDEM tumors as well [4,17,18,19,20,21,31]. It has already been reported that IONM has the ability to accurately predict postoperative deficits [4,18,19,20,21], increases the safety of tumor resection by preventing the occurrence of permanent deficits [17,18], and potentially reduces the postoperative neurological sequelae, which, nevertheless, remains uncertain [31]. Some controversies on the optimal warning criteria exist, which should guide surgical decisions with the optimal balance between falsely positive and falsely negative IONM alerts. Regarding the application of MEP, most authors apply >50% amplitude decrease [4,18,19,20,21,32], >10% latency increase [20,32], or 20% increase in threshold [20] as a criterion imposing temporary surgery stoppage, or its definitive ending in case of persistent MEP amplitude decrease. Contrarily, the adoption of a slightly higher threshold for amplitude decline (being >50–60%) considered as a MEP alert has been reported [31]. Although the threshold > 50% of MEP amplitude decline leads to the risk of false-positive MEP alerts, it appears that such a cutoff point enables optimally balancing false-positive and false-negative alerts [20]. Concurrently, the threshold > 50% of MEP amplitude decline was adopted in our study resulting in the lack of false-positive and false-negative results. Nevertheless, the limited size of our cohort precludes an unequivocal determination of the optimal MEP alert threshold.

It is widely accepted that temporary intraoperative MEP declines, which resolve during the surgery, do not correspond to the occurrence of postoperative neurological deficits [20]. However, our study could not retrospectively identify patients who experienced temporary intraoperative MEP loss, which resolved until the end of surgery. Nevertheless, we can state that all patients with permanent MEP loss had worsening motor function postoperatively. Despite the apparent limitations of our study, we suggest that intraoperative transient > 50% MEP amplitude decline in pediatric IDEM tumor surgery should not impose definitive surgery stoppage, until MEP decrease persists despite the preventive measures that have been taken. Therefore, temporary MEP loss should be considered an important early indicator of the potentially harmful surgical steps, preventing the occurrence of iatrogenic deficits when properly interpreted.

Moreover, considering the high ability of pediatric patients to recover neurologically over time, the long-term accuracy of MEP to predict motor deficits between adults and children is expected to differ. Van der Wal et al. [4] showed a sensitivity of 64% and specificity of 82% of MEP in the short-term assessment following 6-weeks after IDEM tumor surgery. In their long-term evaluation at 1 year, they found an increase in sensitivity to 100% and a concurrent decrease in specificity to 77%. That tendency reflects the transient character of some postoperative deficits, leading to neurological improvement over time. Notably, the study by van der Wal et al. [4] was based mostly on adult patients, which own lower capabilities of neurological recovery than children. Therefore, it would be of great interest to determine the ability of MEP to predict long-term neurological impairment among pediatric patients who underwent IDEM tumor removal. While in our cohort, both patients with significant MEP alterations initially had aggressive tumors, underwent subtotal resection, and died shortly after discharge, our study could not determine the long-term neurological recovery of their deficits.

Furthermore, all recent studies concerning MEP in IDEM tumors indicated a high NPV of 97% [18] and specificity (ranging from 82% to 97%) [4,18,32], which remain closely related to our 100% values. Still, our 100% of PPV and sensitivity remain somewhat higher than reported in the literature (accounting for 66.67% for PPV [18] and ranging from 62.5% to 90% for sensitivity [4,18,32]). Apart from the limited cohort, these disparities could have also resulted from a GTR rate and tumor histology. Since 31.2% of patients underwent subtotal tumor resection, it is presumed that with more frequent GTR, the incidence of MEP alerts would increase, with some of them being false-positive, thus reducing PPV. Moreover, our study included four aggressive tumors whose infiltrative character and poor delineation from the normal tissue precluded GTR.

Notably, both MEP alerts reported in our study occurred in individuals with aggressive tumors. Therefore, apart from the purely predictive value of MEP in IDEM tumor surgery, we believe that IONM can be specifically supportive in poorly delineated aggressive tumors, thus preventing permanent neural injury with the concurrently increased EOR. However, that conclusion cannot be driven based solely on our study and needs to be proved by a larger comparative study. Therefore, we strongly encourage researchers to validate our data based on a larger pediatric IDEM tumor cohort. However, according to the limited literature and our own results, we conclude that MEP can significantly predict the occurrence of postoperative iatrogenic motor deficits with relatively high specificity and NPV. Thereby, negative MEP responses are reflective of uninjured corticospinal tract fibers, allowing to safely proceed with tumor excision, with the minimized risk of iatrogenic motor deficits occurring postoperatively. On the other hand, as reported by Ghadirpour et al. [18], relatively low sensitivity values and PPV necessitate particular caution when MEP changes appear intraoperatively. False-positive MEP alerts can lead to the artificial interruption of surgery, thus limiting the EOR, which might significantly affect the patient’s prognosis. While our study found MEP of 100% sensitivity and PPV, we believe that with a larger cohort, these values might be expected to drop, similar to those presented in Ghadirpour’s study [18].

Concerning MEP sensitivity and PPV, it is essential to address the impact of IONM on the EOR in IDEM tumors. Our study found that IONM alerts significantly affected the EOR. While 78.6% of patients without MEP changes underwent GTR, no patients with persistent MEP amplitude drop of 50% or more underwent GTR (*p* = 0.025). Accordingly, we can conclude that MEP significantly limits the EOR. However, since we found MEP of 100% sensitivity and PPV, the subtotal resections were performed to prevent permanent motor deficits in those patients. However, as stated previously, we were unable to follow-up our patients in order to evaluate the permanence of their postoperative deficits for a longer time period than the time of hospital stay since both patients died shortly after due to the aggressiveness and disseminated character of the tumor. Van der Wal et al. [4], based on their experience with SSEP and MEP, found that overall IONM did not influence the EOR, despite 80% of falsely positive IONM alerts in the GTR group and 20% of alerts in the subtotal resection group. Despite being surprising, these differences could reflect the authors' vast experience with IONM and associated awareness of possible falsely positive IONM changes. Similarly, Cofano et al. [31], in their comparative study with monitored (with MEP, SSEP, and D-wave) and nonmonitored controls, found that the EOR was not associated with overall IONM alerts.

### Limitations

Despite being the first report on the IONM utility in pediatric IDEM tumors, our study has some limitations, which should be thoroughly addressed. Firstly, this is a single-institutional retrospective study on a limited cohort. Moreover, our study did not intend to evaluate SSEP and D-wave monitoring. Merely two patients with significant IONM changes could have artificially overestimated the PPV and sensitivity of MEP, which would probably decrease with the larger cohort and more IONM events. Finally, our IONM data were based on the short-term clinical evaluation, which resulted from the initial aggressiveness of the disease leading to both patients’ death shortly after discharge from the hospital, precluding long-term evaluation of the MEP’s sensitivity, specificity, PPV, NPV and overall accuracy in predicting motor deficits. Therefore, we encourage researchers to validate our data with long-term observation to study the impact of potential neurological recovery on the MEP’s accuracy in predicting long-term motor status.

## 5. Conclusions

MEP accurately predicted postoperative motor deficits. While MEP alerts limited the extent of tumor resection, their high sensitivity and PPV indicate their important role in avoiding iatrogenic motor deficits. Concurrently, high specificity and NPV enable safer further tumor excision with minimized risk of postoperative iatrogenic motor function decline. MEP can be considered a reliable IONM modality to guide surgical decisions in pediatric patients with IDEM tumors. However, our study is based on a limited cohort, with merely two MEP alerts, which might have overestimated true MEP accuracy. We advise caution in the interpretation of our data. Further studies are encouraged to validate our data on a larger pediatric cohort, and additionally evaluate the role of D-wave monitoring in pediatric IDEM tumor surgery.

## Figures and Tables

**Table 1:** Characteristics of the pediatric patients presenting with IDEM tumors.

No.	Sex	Age (Years)	McCormick Grade at Admission	Neurological Deficits	Tumor Location (Vertebral Levels)	Histology	MEP Events	McCormick Grade at Discharge	Postoperative Deficits
1	F	17	1	-	S1-S3, posterolateral	Medulloblastoma (CSF seeding)		1	
2	F	16	1	Sensory	L5-S2, anterior	Myxopapillary ependymoma		1	
3	M	15	2	Motor	T12-L2, L5-S3, posterior	Myxopapillary ependymoma		2	
4	M	9	2	Sensory, motor	L2-L4, lateral	Schwannoma		2	
5	F	5	4	Motor	C4-C7, anterolateral	Rhabdoid meningioma	YES	5	Motor
6	M	6	3	Motor	L5-S2, posterior	Epidermal cyst		3	
7	M	1	2	-	C1-C5, posterolateral	Lipoma		1	
8	F	14	4	Sensory, motor	T12, L4, posterior	Clear cell meningioma		3	
9	M	18	4	Sensory, motor	T12-L2, posterolateral	Myxopapillary ependymoma		4	
10	F	15	1	-	L1-L4, posterior	Ependymoma		1	
11	M	8	5	Motor, urinary incontinence	C2-C4, posterior	NGGCT (CSF seeding)		4	
12	M	7	3	Sensory, motor	C5-T4, posterolateral	Anaplastic ependymoma	YES	4	Motor
13	F	7	5	Sensory, motor, urinary incontinence	T12-L4, posterior	Dermoid cyst		5	
14	F	16	1	-	L3-L5, lateral	Clear cell meningioma		1	
15	F	7	1	-	L4, posterior	Myxopapillary ependymoma		1	
16	M	14	3	Motor	C1-C2, anterolateral	Endodermal cyst		2	

Legend: IDEM, intradural extramedullary; NGGCT, nongerminomatous germ-cell tumor; CSF, cerebrospinal fluid.

## Data Availability

The data generated during this study are available within the article. Datasets analyzed during the current study preparation are available from the corresponding author on reasonable request.
